# ‘Drive the doctor’ for endovascular thrombectomy in a rural area: a simulation study

**DOI:** 10.1186/s12913-023-09672-5

**Published:** 2023-07-20

**Authors:** Willemijn J. Maas, Durk-Jouke van der Zee, Maarten M.H. Lahr, Marc Bouma, Erik Buskens, Maarten Uyttenboogaart

**Affiliations:** 1 Department of Neurology, University of Groningen, University Medical Center Groningen, Groningen, The Netherlands; 2 Health Technology Assessment, Department of Epidemiology, University of Groningen, University Medical Center Groningen, Groningen, The Netherlands; 3 Department of Operations, Faculty of Economics & Business, University of Groningen, Groningen, The Netherlands; 4 Department of Radiology, Medical Imaging Center, University of Groningen, University Medical Center Groningen, Groningen, The Netherlands

**Keywords:** Endovascular thrombectomy, Drive the doctor model, Simulation modeling

## Abstract

**Background:**

Patients who present in a primary stroke center (PSC) with ischemic stroke are usually transferred to a comprehensive stroke center (CSC) in case of a large vessel occlusion (LVO) for endovascular thrombectomy (EVT) treatment, the so-called ‘drip-and-ship’ (DS) model. The ‘drive-the-doctor’ (DD) model modifies the DS model by allowing mobile interventionalists (MIs) to transfer to an upgraded PSC acting as a thrombectomy capable stroke center (TSC), instead of transferring patients to a CSC. Using simulation we estimated time savings and impact on clinical outcome of DD in a rural region.

**Methods:**

Data from EVT patients in northern Netherlands was prospectively collected in the MR CLEAN Registry between July 2014 - November 2017. A Monte Carlo simulation model of DS patients served as baseline model. Scenarios included regional spread of TSCs, pre-hospital patient routing to ‘the nearest PSC’ or ‘nearest TSC’, MI’s notification after LVO confirmation or earlier prehospital, and MI’s transport modalities. Primary outcomes are onset to groin puncture (OTG) and predicted probability of favorable outcome (PPFO) (mRS 0–2).

**Results:**

Combining all scenarios OTG would be reduced by 28–58 min and PPFO would be increased by 3.4-7.1%. Best performing and acceptable scenario was a combination of 3 TSCs, prehospital patient routing based on the RACE scale, MI notification after LVO confirmation and MI’s transfer by ambulance. OTG would reduce by 48 min and PPFO would increase by 5.9%.

**Conclusions:**

A DD model is a feasible scenario to optimize acute stroke services for EVT eligible patients in rural regions. Key design decisions in implementing the DD model for a specific region are regional spread of TSCs, patient routing strategy, and MI’s notification moment and transport modality.

**Supplementary Information:**

The online version contains supplementary material available at 10.1186/s12913-023-09672-5.

## Background

Timing of intravenous thrombolysis (IVT) and endovascular thrombectomy (EVT) is crucial for patients with acute ischemic stroke due to large vessel occlusion (LVO). The sooner the patient receives appropriate treatment, the higher the probability of a favorable outcome after 90 days. [1–3].

Historically, two dominant organizational models emerged routing LVO patients towards EVT capable centers. Routing patients directly to a comprehensive stroke center (CSC) for IVT and EVT, is defined as the ‘mothership’ (MS) model. Patients routed to a primary stroke center (PSC), possibly followed by a second transfer to a CSC for EVT, is defined as the ‘drip-and-ship’ (DS) model. Inter-hospital transfer within the DS model contributes substantially to the delay from stroke onset to groin puncture (OTG) [4–6].

Currently, the geographic locations of stroke onset and the nearest center influence the decision of which organizational model, DS or MS, is used for routing LVO patients [7]. An alternative to the DS model, is the ‘drive-the-doctor’ (DD) model [8–11]. The DD model seeks to reduce OTG excluding inter-hospital delay for LVO patients. Implementing the DD model assumes upgrading selected PSCs to thrombectomy capable stroke centers (TSCs), [10] and relies on mobile neuro-interventionalists (MIs) from a nearby CSC to travel to the TSC. Previous studies show that OTG times for DD and MS models are comparable [10] and, more importantly, shorter than OTG times for the DS model [11]. Early notification of the MI is paramount. Ideally, LVO would be assessed and confirmed at the stroke onset location. Both a timely arrival of the MI, as well as direct routing of former DS patients to a TSC – for whom the nearest PSC has not been upgraded to a TSC – would shorten the OTG. Pre-hospital scales may be helpful in notifying MI and routing patients. Currently, the predictive value of pre-hospital stroke scales to detect LVO patients is acceptable-to-good. However, their impact on patient routing remains unclear [12].

Although the DD model has shown promising results in urban regions, less is known about the generalizability to rural areas [13]. To study the potential of the DD model within a certain region, region-specific characteristics should be taken into account such as the health infrastructure (spread of PSCs/CSCs), patient routing strategy based on pre-hospital triage scales or not, and pathway set-up in terms of services that might affect workflow efficiency, both for patients and MIs [14]. A well-established methodology to study the organizational set-up of the acute stroke pathway is simulation modeling [15–17]. With our modeling study we aim to study the potential impact of the DD model in the northern Netherlands rural region.

## Methods

### Participants

Prospectively collected data of 183 DS patients from the MR CLEAN Registry [18] was used as input for the baseline model. Patients were treated with EVT between July 2014 and November 2017 in the University Medical Center Groningen (UMCG). For a complete overview of all time delays along the acute stroke pathway for each patient, hospital data was supplemented with retrospectively collected data of Emergency Medical Services (EMSs) [19]. Exclusion criteria were pre-stroke modified Rankin scale (mRS) score > 2 or OTG time > 390 min. Longer OTG was excluded because perfusion imaging guided EVT beyond 6 h was not standard of care during that time period.

### Setting

**Fig. 1:**
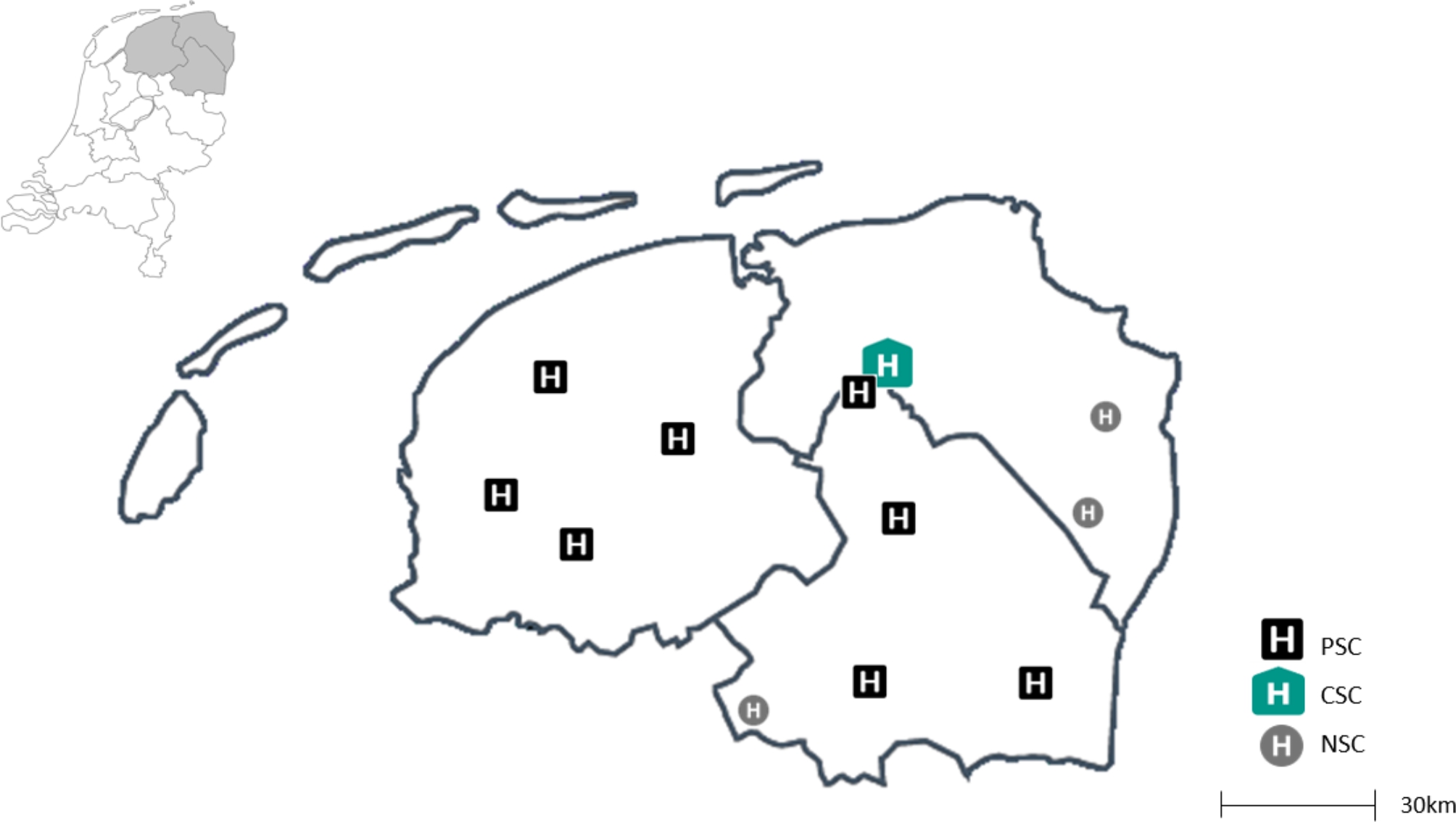
Primary Stroke Centers (PSCs), Comprehensive Stroke Center (CSC) and Non Stroke Centers (NSCs) in the north of the Netherlands PSC, primary stroke center; CSC, comprehensive stroke center; NSC, non-stroke center

Our rural region is served by a single CSC, the UMCG, and eight PSCs at distances ranging from 6 to 84 km from the CSC (Fig. 1). The catchment area includes a population size of around 1.7 million (209 inhabitants per square kilometer). All PSCs and the CSC have state of the art stroke units with 24/7 availability of a neurologist.

### Baseline model

A Monte Carlo simulation model was developed based on the collected data. Plant Simulation^TM,^ [20] software was used to build the model. Recorded time intervals for each step along the acute stroke pathway were used to generate time distributions as input for the baseline model. The following time variables were used: symptom onset or last seen well, time of 911 call, EMS arrival at the stroke onset location, EMS departure to PSC, EMS arrival at PSC, computed tomography (CT), start IVT at PSC, CT angiography (CTA), transfer notification (second 911 call), EMS arrival at PSC, EMS departure to CSC, EMS arrival at the CSC, arrival at angiography suite and groin puncture. ExpertFit^TM,^ [21] was used to obtain distributions for the simulation model. Missing values were excluded from analyses, as statistical imputation techniques were not necessary to obtain intact distributions for building the simulation model. Time lapses of the baseline model were numerically validated by comparing the model output (mean, median, standard deviation, minimum and maximum) with real-world data of the patients.

To assess the effect of time delays on patient outcome, predictions for each of the 7- levels of mRS were obtained by ordinal logistic regression. Prognostic variables that were used included: OTG (continuous), age (continuous), National Institutes of Health Stroke Scale score (continuous), and CTA collateral grading score in 4 categories (absent of collaterals, less than 50% filling of occluded area, more than 50% filling but less than 100% filling of occluded area or 100% filling of occluded area). Formulas obtained by ordinal regression were used to estimate the predicted probability of favorable outcome (PPFO) (mRS 0–2) within the simulation model. Supplementary material of another simulation modeling [22] study provides more detail on the development of the baseline model.

### Construction of DD model

To study the effects on time to treatment and clinical outcomes with the hypothetical implementation of the DD model in our rural region, the baseline model was modified based on input from literature review, expert opinion and several data sources for transport times and other time variables. As a result of introducing the DD model the activities and pathway set-up for both the MI and patient will change, including new variables and time metrics [13].

New variables identified for MI’s transport to the TSC are: (1) MI’s dispatch timing from the CSC and (2) the MI-to-TSC transport time [13]. Several studies define MI’s notification moment after LVO confirmation [9–11, 13, 23]. Assuming this moment will be directly after CTA, this moment is modeled as MI’s notification. For estimating the MI-to-TSC transport time, MI’s start location is an important variable and described in the literature as close to or in the CSC [24]. In addition, the choice of transport modality influences treatment delays. We assumed that MI started transport from the CSC. In addition, car, ground EMS (GEMS) and helicopter EMS (HEMS) are possible transport modalities options in our region. To model MI’s path as realistic as possible, a time delay from notification moment to departure was added (5 to 10 min, i.e. 10 min when MI views the CTA through telemedicine as preparation for EVT). Likewise, MI time from TSC arrival to angiography suite arrival was estimated at 5 or 10 min, depending on the location of hospital landing areas.

Parallel to MI’s path, and following LVO confirmation, the time path for patients from CTA to angiography suite in the TSC will change accordingly, i.e. the patient will be prepared for EVT at the TSC [11, 24]. Supplementary material, Tables S1 and S2, provides more details about the used distributions for constructing the DD model.

### Data sources used for DD construction

Several data sources were used for DD modeling. For car transport of MI, distributions were generated by car travel times from CSC postal code to TSC postal codes, using a web-based route planner (https://www.anwb.nl/verkeer/routeplanner) [25]. Modeling GEMS is based on data collected at regional EMSs. HEMS data was collected at the ANWB Medical Air Assistance, which is the operator of helicopters for medical purposes in the Netherlands. To model patient time intervals from CTA to angiography arrival in the TSC data was used from the MR CLEAN Registry [18] on all MS patients treated with EVT in the Netherlands between November 2016 and November 2017.

#### Scenarios

**Fig. 2:**
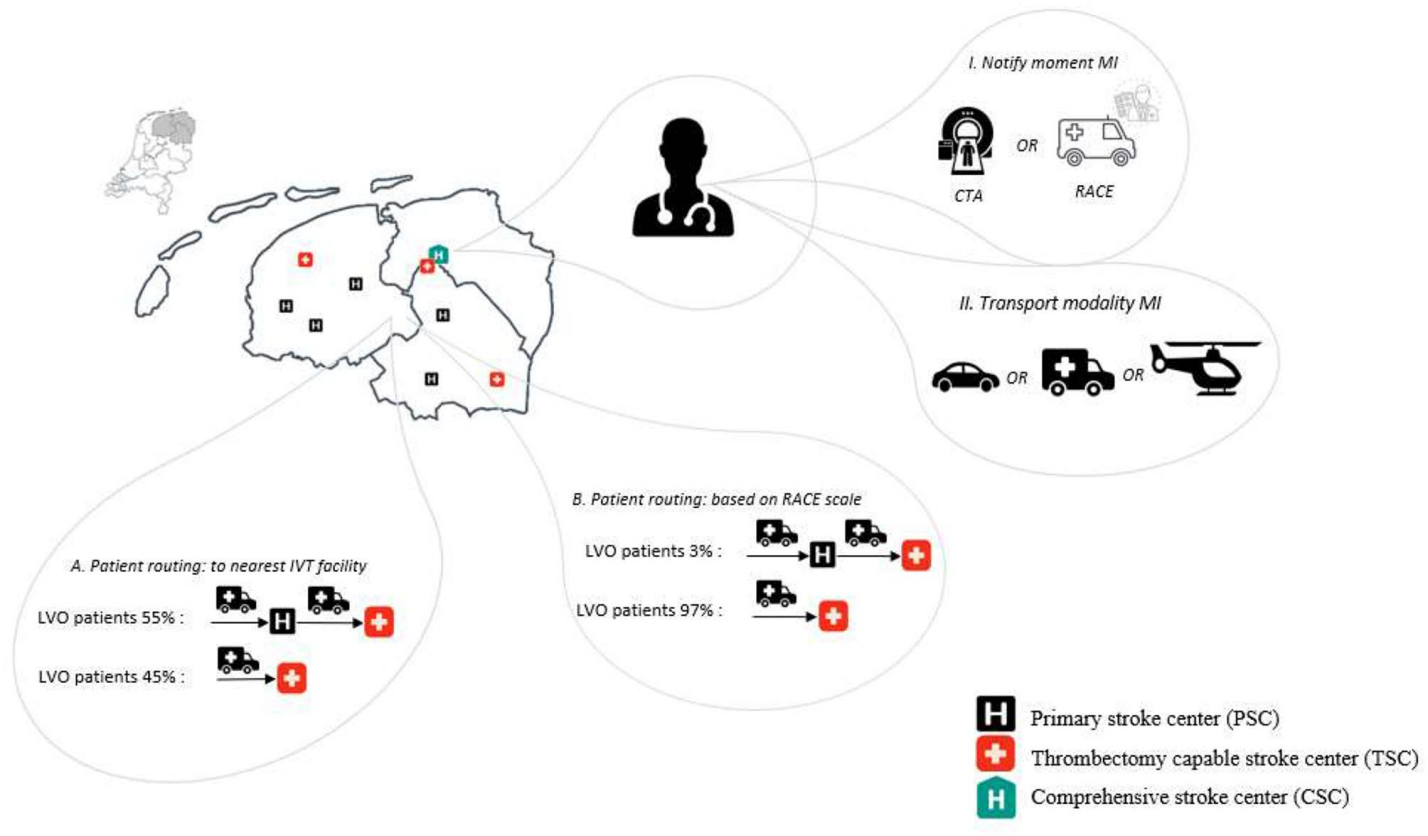
Scenarios for modeling ‘drive the doctor’ in northern Netherlands MI, Mobile interventionalist; IVT, intravenous thrombolysis; RACE, rapid arterial occlusion evaluation; LVO, large vessel occlusion • Spread of TSCs level, 3TSCs • Patient routing level; (A) to nearest IVT facility, (B) based on RACE scale • Workflow level; (I) Choices of MI notify moment, after CTA when LVO is confirmed or when paramedic on scene indicates ‘yes’ for LVO, based on RACE scale, (II) MI transport modality, Car, Ground EMS (GEMS) and Helicopter EMS (HEMS).

For the hypothetical implementation of the DD model, several design decisions were taken into account in developing scenarios. Design decisions relate to: (1) regional spread of TSCs, (2) patient routing strategy and (3) workflow of MI’s path (Fig. 2).

### Regional spread of TSCs

Within our region, based on available resources, available supporting staff and expected treatment volumes, [26] upgrading three existing PSCs to TSCs was considered a realistic scenario and subsequently modeled.

### Patient routing strategy

The first routing scenario was based on local guidelines, i.e., pre-hospital patient routing to the nearest IVT-capable hospital. This scenario will yield two types of patient routing: (1) patients routed directly to a TSC and (2) combined DS/DD, implying patient routing to the nearest PSC and next to the nearest TSC.

In addition, an alternative routing strategy is modeled based on a pre-hospital rapid arterial occlusion evaluation (RACE) scale [27]. When LVO is suspected patients will be routed to the nearest TSC. The PRE-hospital Stroke Treatment Organization (PRESTO) study [12] showed a negative predictive value of 95% which means that only 5% of all LVO patients will not directly be transported to a TSC. This latter percentage is therefore still modeled as DS/DD patients. Patients located closest to a TSC (45% of all DS patients collected in our study) will be routed to the nearest TSC irrespective of their score on the RACE scale. Therefore, the percentage of LVO patients routed to the nearest PSC (i.e. DS/DD routing) is 3% instead of 5%, (55% of 5% = approximately 3%) (Fig. 2, scenario A and B).

### Workflow of MI’s path

There are several options to influence the workflow of MI’s path when hypothetically implementing the DD model: (1) MI’s notification moment to travel to a TSC, and (2) the choice of transport modalities for MI (Fig. 2, scenario I and II).

Based on the literature, the moment directly after CTA has been chosen as the first option for the notification moment [9–11, 13, 23, 24]. An alternative notification moment is based on the use of the RACE scale that is executed on scene by paramedics. Based on the negative predictive value of the RACE scale (95%), in 5% of the LVO cases, MI was not notified by EMS paramedics. For these situations, we modeled that MI was notified after LVO confirmation with CTA.

Transport modality options modeled in our region were car (baseline DD), GEMS and HEMS (Fig. 2). For transport by car we assumed that MI departs at the CSC. For GEMS we assumed that the ambulance had to come from the ambulance station and therefore, a median (IQR) ambulance response time of 9 min (7–12) (based on the collected EMS data) was included within MI’s path. HEMS is stationed near the city of Groningen and has to pick up MI from the CSC. Parallel to this time a fire safety team has to be present on the helipad of the CSC, which takes 20 min, and the MI has to be picked up (4 min). Therefore an extra time of 24 min has been included, in addition to the time needed to fly from CSC to a TSC. 15% of the time GEMS was used in the HEMS scenario due to bad weather conditions. GEMS and HEMS response times are modeled in parallel with MI’s preparing time to leave (5 to 10 min, depending on a CTA telemedicine preparation for EVT or not).

### Sensitivity analysis

Because no data was available for the time interval between CTA to angiography suite arrival in the hypothetical TSC, we performed sensitivity analyses. This sensitivity analysis was based on the distribution for time from CTA to angiography arrival within hospitals and therefore, the patient preparing time was 25% longer or shorter compared to the mean value for all MS patients of the MR CLEAN Registry [18] treated with EVT between November 2016 and November 2017.

### Outcome measures and statistics

For each scenario, we calculated the clinical benefits in terms of reduction in OTG and the PPFO compared to the baseline model. Significance testing was inapplicable, as the goal is to assess the potential gain expected based on 100,000 hypothetical patients rather than to test a hypothesis in an actual experiment.

Secondary outcomes are the mean times of patients waiting for the MI and vice versa (i.e. waiting time). In addition, the percentage of patients who have to wait for MI arrival and the percentage of MIs that have to wait for the patient were calculated.

## Results

### Baseline characteristics

One hundred and sixty five patients met the inclusion criteria. Fourteen patients were excluded because of an unknown pre-stroke mRS or > 2 and 4 patients because of an OTG > 390 min. Data on clinical characteristics, diagnostics processes and time delay variables that served as input for the DS baseline model are presented in a previous published study [22] and included in the supplementary material, Table S3.

#### Simulation results

Table 1 shows the results of all scenarios that were simulated, in terms of OTG, PPFO and patient / MI waiting times. Results of MI-to-TSC transport time per transport modality are shown in the supplementary material.

**Table 1:** Results of modeling DD in our region

Infrastructure	Routing strategy	Notify moment MI	Transport modality	OTG (95% CI)	PPFO (95% CI)	Waiting time MI in minutes (percentage*)	Waiting time patient in minutes (percentage*)
Baseline	Baseline	Baseline	Baseline	241.1 (240.7–241.4)	52.4 (52.2–52.5)	NA	NA
3 TSCs	to nearest IVT facility	CTA	Car	212.9 (212.5–213.3)	55.8 (55.7–55.9)	33.1 (72)	6.8 (28)
			GEMS	210.2 (209.8–210.6)	56.1 (56.0–56.3)	40.3 (78)	4.1 (22)
			HEMS	210.3 (209.9–210.7)	56.1 (56.0–56.3)	40.2 (76)	4.2 (24)
		RACE scale on scene	Car	207.5 (207.1–207.9)	56.5 (56.3–56.6)	67.9 (92)	1.4 (8)
			GEMS	206.8 (206.4–207.2)	56.5 (56.4–56.7)	77.2 (95)	0.7 (5)
			HEMS	206.8 (206.4–207.2)	56.6 (56.4–56.7)	76.9 (95)	0.7 (5)
	RACE scale on scene	CTA	Car	199.2 (198.8–199.5)	57.5 (57.4–57.6)	10.7 (37)	16.5 (63)
			GEMS	192.7 (192.4–193.0)	58.3 (58.2–58.4)	14.2 (49)	10.0 (51)
			HEMS	192.3 (192.0–192.6)	58.4 (58.2–58.5)	13.5 (47)	9.6 (53)
		RACE scale on scene	Car	183.8 (183.5–184.2)	59.4 (59.2–59.5)	44.5 (90)	1.1 (10)
			GEMS	183.1 (182.8–183.5)	59.5 (59.3–59.6)	53.7 (96)	0.4 (4)
			HEMS	183.0 (182.7–183.4)	59.5 (59.3–59.6)	53.4 (97)	0.3 (3)

OTG, onset to groin puncture; CI, confidence interval; PPFO, predicted probability of favorable outcome; MI, Mobile interventionalist; TSC, Thrombectomy capable stroke center; CTA, computed tomography angiography; GEMS, ground emergency medical services; HEMS, helicopter emergency medical services; RACE, rapid arterial occlusion evaluation.*percentage at which MI or patient has to wait for the other

When hypothetically implementing the DD model with three TSCs and making conservative design decisions with respect to patient routing and set-up of MI’s path, i.e. patient routing to the nearest IVT facility, MI notification moment after CTA and car as MI transport modality, OTG would be reduced by 28 min and PPFO would increase by 3.4% compared to the baseline model.

When combining the introduction of three TSCs with optimal design decisions on patient routing and set-up of MI path, meaning the use the RACE scale in patient routing, notify MI after using RACE scale on scene, and HEMS as a transport modality, OTG would be reduced by 58 min and PPFO would increase by 7.1% compared to the baseline model. This latter approach would imply mean MI waiting times of 53 min, while having to wait in 97% of the cases. A scenario fostering good use of staff and resources may be related to patient routing strategy based on RACE scale, MI’s notification moment after CTA and GEMS being used as a transport modality. Compared to the baseline, OTG could be reduced by 48 min and PPFO increased by 5.9%. Waiting patterns for patients vs. MIs are rather similar in terms of their mean waiting times, 10 vs. 14 min, and need to wait, 51% vs. 49%.

### Sensitivity analysis

Table S4 and S5 of the supplementary material shows results of the sensitivity analysis. When the patient preparation time (CTA to angiography suite arrival) was 25% shorter than the benchmark variable (i.e. 45 min), OTG may be reduced by 4–17 min and PPFO will increase by 0.5-2.0%. Differences in results depend on design decisions made in implementing DD. Additionally, when patient preparation time was 25% longer compared to the benchmark variable, OTG will increase by 1–10 min, while PPFO will decrease by minus 0.1–1.2%.

## Discussion

To our knowledge, this is the first simulation modeling study, based on patient level data, analyzing and designing the hypothetical introduction of the DD model in a rural region. It extends previous studies relying on conditional probability models, [28, 29] by allowing for greater precision in estimating OTG and outcomes. Results show that implementation of the DD model goes together with design efforts, seeking the best combination of regional characteristics, design options and consequences for resource use. All scenarios studied indicated clear benefits of implementing a DD model in our region, with reductions of OTG ranging from 28 to 58 min and PPFO increasing 3.4–7.1% at maximum. The most optimal results were found for the fastest scenario setting little restrictions to resource use, i.e., patient routing based on RACE scale, MI’s notification on onset scene based on RACE and, MI’s transport by HEMS. This approach would, however, be unacceptable because in this scenario MI will be many times alerted and will travel for patients that finally do not have a LVO. Also, using HEMS, with times compared to GEMS, is probably not cost-effective to implement in our region. The scenario of MI notification after CTA and transportation by GEMS would realize most of the gains, i.e., OTG being reduced by 48 min and PPFO being increased by 5.9%, with an acceptable burden for the MI.

When implementing the DD model in a certain region, urban or rural, the first design decision is based on which PSCs are suitable for upgrading to a TSC. Available resources, for example, a suitable angiography suite, available supporting staff and expected treatment volumes [26] are important in making a decision on which PSC is suitable. In addition, post interventional care in our region is well organized in all PSCs (24/7 availability of a neurologist and available rehabilitation therapies), but for other regions, this might play a role in the choice of upgrading a PSC. Furthermore, the distributions of TSCs within a region is important, meaning their location relative to other TSCs and CSC(s). If a suitable PSC is very close to a CSC (e.g. within 15 min), the option of bypassing a PSC may be better [30] as patients routed according to the MS model seem to have a faster OTG or comparable to the DD model [10, 11]. More distant PSCs are a good option for upgrading to a TSC, also taking into account the time to IVT for non LVO patients. In addition, setting up advanced stroke facilities for EVT in centers near to each other may require further research on costs and cost-effectiveness.

Furthermore, in our model we adapted the current patient routing strategy, i.e., transport stroke patients to the nearest IVT-capable center, using the RACE scale in decision making instead. However, many alternative means for supporting routing decisions are available or under development, like the Gaze-Face-Arm-Speech-Time (G-FAST) and the Conveniently-Grasped Field Assessment Stroke Triage (CG-FAST) scales [12], telemedicine, and mobile stroke units.

Workflow efficiency for patients and/or MIs are important considerations in making DD a success. Choice of transport modality seems to be an important determinant in optimizing MI’s path. In rural areas with long interhospital distances, HEMS are more feasible than GEMS or transport by car [31]. Urban areas may allow for MI traveling by a taxi, [9] subway [13] or other public transport [23]. Likewise, preparing time in the TSC does matter for patient outcomes. As these time intervals were obviously not available for the hypothetical DD model, we estimated these time intervals based on data from MR CLEAN Registry. Sensitivity analysis on preparing times illustrate how they have a relevant impact on OTG and patient outcomes (Table S2). Therefore, in addition to optimizing workflow for MIs, it is important that the patient workflow in the TSC is optimized as well so that the delay will be minimal towards EVT.

### Limitations

Our study has limitations. Our Monte Carlo simulation model included only data from LVO patients that were treated with EVT. However, acute stroke care also concerns IVT eligible patients without EVT, hemorrhage stroke patients and stroke mimics. A simulation model that includes all suspected stroke patients would provide a more realistic picture of how a DD model will affect acute stroke care. By using such a simulation model, the effect on onset to IVT for patients not eligible for EVT could be estimated, for example, when routing non LVO patients to a more distant TSC instead of the nearest PSC.

Furthermore, implementation issues faced in setting up a well operating stroke team, hosting the MI and local staff, and EVT facilities at the TSC in terms of potential effects on delays and outcomes have not been considered in this study. Yet, a previous study showed no significant difference in results between patients treated in a PSC or CSC [31]. Nevertheless, further research may be required on implementation issues faced when upgrading a PSC to a TSC to guarantee DD being feasible.

## Conclusions

A DD model is a feasible scenario to optimize acute stroke services for EVT eligible patients in rural regions. Key design decisions in implementing the DD model for a specific region are regional spread of TSCs, patient routing strategy, and MI’s notification moment and transport modality.

## Electronic supplementary material

Below is the link to the electronic supplementary material.

Supplementary Material 1

## Data Availability

The source data for this sub-study are obtained from the MR CLEAN Registry, which is not an open source. Likewise, the source data of the EMS are not publicly available, as these would enable the identification of individual centres. The sharing of such data is in conflict with the privacy regulations in the Netherlands. However, all data generated and data distributions underlying the simulation model are available for scrutiny and re-use, and are included in this published article and its supplementary information files.
