# Supplementary material for: ‘Drive the doctor’ for endovascular thrombectomy in a rural area: a simulation study
Source: BMC Health Serv Res. 2023 Jul 20;23:778. doi: 10.1186/s12913-023-09672-5 (PMC10360278; doi:10.1186/s12913-023-09672-5)
Supplement: Supplementary file 1 — Supplementary Material 1 [file 12913_2023_9672_MOESM1_ESM.docx]

**Supplementary material;** ‘Drive the doctor’ for endovascular thrombectomy in a rural area: a simulation study.

**Introduction**

The main text of the manuscript provides the most important findings of the study. This supplementary material provides details on the construction of the ‘drive the doctor’ (DD) model, results of used data and results of the sensitivity analysis.

Details about the distributions of the baseline, drip and ship (DS) model, can be found in the supplemental material of ‘Expediting workflow in the acute stroke pathway for endovascular thrombectomy in the northern Netherlands: a simulation model.’^1^ Note that the respective model has been slightly adapted to suit the aims of the current study. Changes concern the representation of EMS transfers from regional PSCs to the three newly defined TSCs. Therefore the step ‘EMS transfer’ of the interhospital transfer is divided in three categories, western part of our region, north eastern part of our region and the south eastern part of our region, i.e., the catchment areas of the new TCSs. Distributions are as follow; Beta (Lower endpoint = 17.92; Upper endpoint = 43.91; α1 = 1.24; α2 = 1.85), Lognormal (μ = 10.97; σ = 7.03), Log-logistic (Location = 25.02; α = 7.46; β = 3.80), respectively.

**Construction of DD model: input parameters**

Table S1 shows the used distributions for constructing the DD model. First the distribution of patients treated at a certain TSC is shown. For use of car, ground emergency medical service (GEMS) and helicopter EMS (HEMS) distributions are different as shown in the table. The distribution of time from Mobile interventionalists (MI’s) arrival at the thrombectomy capable stroke center (TSC) to the angiography suite is assumed to be the same for all transport modalities.

Table S2 shows the used distributions for the preparation of the patients in the TSC after CTA, i.e. time from CTA to angiography suite. The distributions differ per emergency department (ED) route.^1^

Table S1; input parameters to construct DD model

| Activity duration | Distribution | Parameters | | |
| --- | --- | --- | --- | --- |
| TSC: | Value | Frequency / Time | | |
|  | TSC 1 | 31 | | |
|  | TSC 2 | 15 | | |
|  | TSC 3 | 27 | | |
| **Car** |  |  | | |
| Departure time | Uniform | a = 5.00; b = 10.00 | | |
| Transport time TSC 1 | Constant | 48 | | |
| Transport time TSC 2 | Constant | 54 | | |
| Transport time TSC 3 | Constant | 13 | | |
| **Ground EMS (GEMS):** |  |  | | |
| Response time EMS | Continuous empirical | Lower Bound | Upper Bound | Frequency |
|  |  | 0 | 2 | 12 |
|  |  | 2 | 4 | 17 |
|  |  | 4 | 6 | 18 |
|  |  | 6 | 8 | 29 |
|  |  | 8 | 10 | 39 |
|  |  | 10 | 15 | 17 |
|  |  | 15 | 30 | 8 |
| Departure time (time for MI to step into the ambulance) | Constant | 5 |  |  |
| Transport time TSC 1 | Beta | Lower endpoint = 17.92; Upper endpoint = 43.91; α1 = 1.24; α2 = 1.85 | | |
| Transport time TSC 2 | Log-logistic | Location = 25.02; α = 7.46; β = 3.80 | | |
| Transport time TSC 3 | Lognormal | μ = 10.97; σ = 7.03 | | |
| **Helicopter EMS (HEMS):** |  |  | | |
| Departure time + response HEMS, parallel fire safety team present and picking up MI | Constant | 24 | | |
| Transport time TSC 1 | Uniform | a = 14.00; b = 18.00 | | |
| Transport time TSC 2 | Uniform | a = 15.00; b = 19.00 | | |
| Transport time TSC 3 | Uniform | a = 2.00; b = 2.00 | | |
| MI’s arrival to angiography suite TSC1 | Constant | 10 | | |
| MI’s arrival to angiography suite TSC2 or TSC3 | Constant | 5 | | |

Table S2; input parameters for patient preparing time when patient is directly routed to a TSC; CTA to angiography suite based on ED routing.

| Activity duration | Distribution | Parameters |
| --- | --- | --- |
| Preparation patient route 1 | Gamma | Location = 4.51; α = 1.99; β = 16.90 |
| Preparation patient route 2 | Gamma | Location = 0.00; α = 2.38; β = 16.05 |
| Preparation patient route 3 | Gamma | Location = 0.87; α = 2.57; β = 18.54 |

**Results**

*Collected data*

Table S3 shows clinical characteristics, diagnostics processes and time delay variables that served as input for the baseline model.

Table S3. Characteristics, diagnostics and time delays of the baseline model.

| *Patient characteristics* |  | N |
| --- | --- | --- |
| Age in years (SD) | 70 (13) | 165 |
| Male (%) | 99 (60) | 165 |
| IVT rate (%) | 132 (80) | 165 |
|  |  |  |
| *Patient diagnostics* |  |  |
| Baseline NIHSS score (IQR) | 17 (12-19) | 164 |
| Collaterals absent (%) | 11 (7) | 155 |
| < 50% filling of collaterals (%) | 81 (49) | 155 |
| > 50% filling of collaterals, less than 100% (%) | 49 (30) | 155 |
|  |  |  |
| *Process times EMS* |  |  |
| Symptom onset to 911 call | 11 (3-33) | 139 |
| Response time | 9 (7-12) | 132 |
| On scene time | 16 (12-20) | 126 |
| Transport time | 12 (7-15) | 122 |
|  |  |  |
| *Process times in-hospital, PSC* |  |  |
| Hospital arrival to CT | 15 (11-20) | 125 |
| CT to EMS call for transfer (including CTA and IVT) | 48 (33-69) | 165 |
|  |  |  |
| *Process times inter-hospital transfer* |  |  |
| Response time | 8 (5-10) | 140 |
| Handover time | 14 (10-16) | 139 |
| Transport time | 27 (19-32) | 150 |
|  |  |  |
| *Process times in-hospital CSC* |  |  |
| CSC arrival to additional diagnostics* | 23 (17-45) | 17 |
| Additional diagnostics* to angiography suite | 29 (14-70) | 18 |
| CSC arrival to angiography suite | 26 (16-38) | 151 |
| Arrival angiography suite to groin | 30 (24-35) | 163 |
|  |  |  |
| *Overall time* |  |  |
| OTG | 230 (198-275) | 165 |

Time variables are in minutes, median (IQR). SD indicates standard deviation; IVT, intravenous thrombolysis; NIHSS, National Institutes of Health Stroke Scale; EMS, Emergency Medical Services; PSC, Primary Stroke Center; CSC, Comprehensive Stroke Center; CT, computed tomography; CTA, computed tomography angiography OTG, time from stroke onset to groin puncture. *for example repeated imaging in case of neurological improvement/deterioration.

*Input data*

The MI-to-TSC transport time per transport modality are as follows: when MI’s transport modality is a car, GEMS or HEMS, the mean (SD) MI-to-TSC transport time are 40.4 (15.5), 22.1 (12.7), and 12.8 (6.1) minutes from CSC to TSC respectively. The median (IQR) CTA to angiography arrival time, i.e. patient preparing time, is 45 (29-62) minutes.

*Sensitivity analysis*

Table S3 and S4 show the results of the sensitivity analysis concerning a 25% faster or slower patient preparing time in the TSC, i.e. time from CTA to angiography suite arrival.

Table S4; patient preparing time (CTA to angiography suite arrival) 25% faster.

| Infrastructure | Routing strategy | Notify moment MI | Transport modality | OTG (95% CI) | PPFO (95% CI) | Waiting time MI (percentage*) | Waiting time patient (percentage*) |
| --- | --- | --- | --- | --- | --- | --- | --- |
| Baseline | Baseline | Baseline | Baseline | 241.1 (240.7 - 241.4) | 52.4 (52.2 - 52.5) | NA | NA |
| 3 TSCs | to nearest IVT facility | CTA | Car | 208.6 (208.2 - 209.0) | 56.3 (56.2 - 56.5) | 28.7 (63) | 10.3 (37) |
|  |  |  | GEMS | 204.9 (204.5 - 205.3) | 56.8 (56.7 - 56.9) | 35.0 (66) | 6.6 (34) |
|  |  |  | HEMS | 205.2 (204.8 - 205.6) | 56.7 (56.6 - 56.9) | 35.1 (61) | 6.9 (39) |
|  |  | RACE scale on scene | Car | 200.4 (199.9 - 200.8) | 57.3 (57.2 - 57.5) | 60.7 (88) | 2.0 (12) |
|  |  |  | GEMS | 199.4 (199.0 - 199.8) | 57.4 (57.3 - 57.6) | 69.7 (93) | 1.0 (7) |
|  |  |  | HEMS | 199.4 (198.9 - 199.8) | 57.4 (57.3 - 57.6) | 69.5 (93) | 1.0 (7) |
|  | RACE scale on scene | CTA | Car | 191.2 (190.9 - 191.5) | 58.5 (58.4 - 58.6) | 2.7 (17) | 25.2 (83) |
|  |  |  | GEMS | 182.1 (181.8 - 182.4) | 59.6 (59.5 - 59.7) | 3.7 (21) | 16.2 (79) |
|  |  |  | HEMS | 181.8 (181.5 - 182.1) | 59.5 (59.4 - 59.6) | 3.1 (14) | 15.9 (86) |
|  |  | RACE scale on scene | Car | 167.3 (167.0 - 167.6) | 61.4 (61.3 - 61.5) | 27.9 (86) | 1.4 (14) |
|  |  |  | GEMS | 166.4 (166.1 - 166.7) | 61.5 (61.4 - 61.6) | 37.0 (96) | 0.5 (4) |
|  |  |  | HEMS | 166.3 (166.0 - 166.6) | 61.5 (61.4 - 61.6) | 36.7 (97) | 0.4 (3) |

OTG, onset to groin puncture; CI, confidence interval; PPFO, predicted probability of favorable outcome; MI, Mobile interventionalist; TSC, Thrombectomy capable stroke center; CTA, computed tomography angiography; GEMS, ground emergency medical services; HEMS, helicopter emergency medical services; RACE, rapid arterial occlusion evaluation.*percentage at which MI or patient has to wait for the other.

Table S5; patient preparing time (CTA to angiography suite arrival) 25% slower.

| Infrastructure | Routing strategy | Notify moment MI | Transport modality | OTG (95% CI) | PPFO(95% CI) | Waiting time MI (percentage*) | Waiting time patient (percentage*) |
| --- | --- | --- | --- | --- | --- | --- | --- |
| Baseline | Baseline | Baseline | Baseline | 241.1 (240.7 - 241.4) | 52.4 (52.2 - 52.5) | NA | NA |
| 3 TSCs | to nearest IVT facility | CTA | Car | 213.4 (213.0 - 213.7) | 55.8 (55.6 - 55.9) | 33.5 (77) | 2.5 (23) |
|  |  |  | GEMS | 211.3 (211.0 - 211.7) | 56.0 (55.9 - 56.1) | 41.5 (91) | 0.5 (9) |
|  |  |  | HEMS | 211.1 (210.7 - 211.5) | 56.0 (55.9 - 56.2) | 41.0 (91) | 0.2 (9) |
|  |  | RACE scale on scene | Car | 211.2 (210.8 - 211.6) | 56.0 (55.9 - 56.2) | 71.6 (97) | 0.1 (3) |
|  |  |  | GEMS | 210.9 (210.6 - 211.3) | 56.1 (55.9 - 56.2) | 81.3 (99) | 0.0 (1) |
|  |  |  | HEMS | 210.9 (210.5 - 211.3) | 56.1 (55.9 - 56.2) | 81.0 (99) | 0.0 (1) |
|  | RACE scale on scene | CTA | Car | 198.6 (198.3 - 198.9) | 57.6 (57.5 - 57.7) | 10.2 (49) | 5.7 (51) |
|  |  |  | GEMS | 193.9 (193.6 - 194.2) | 58.2 (58.0 - 58.3) | 15.5 (80) | 1.0 (20) |
|  |  |  | HEMS | 193.3 (193.0 - 193.6) | 58.2 (58.0 - 58.3) | 14.6 (86) | 0.4 (14) |
|  |  | RACE scale on scene | Car | 193.1 (192.8 - 193.3) | 58.3 (58.1 - 58.4) | 53.7 (99) | 0.1 (1) |
|  |  |  | GEMS | 192.9 (192.6 - 193.2) | 58.3 (58.2 - 58.4) | 63.5 (100) | 0.0 (0) |
|  |  |  | HEMS | 192.9 (192.6 - 193.2) | 58.3 (58.2 - 58.4) | 63.3 (100) | 0.0 (0) |

OTG, onset to groin puncture; CI, confidence interval; PPFO, predicted probability of favorable outcome; MI, Mobile interventionalist; TSC, Thrombectomy capable stroke center; CTA, computed tomography angiography; GEMS, ground emergency medical services; HEMS, helicopter emergency medical services; RACE, rapid arterial occlusion evaluation.*percentage at which MI or patient has to wait for the other.
